# Author Correction: Decoupling PER phosphorylation, stability and rhythmic expression from circadian clock function by abolishing PER-CK1 interaction

**DOI:** 10.1038/s41467-022-32668-4

**Published:** 2022-08-18

**Authors:** Yang An, Baoshi Yuan, Pancheng Xie, Yue Gu, Zhiwei Liu, Tao Wang, Zhihao Li, Ying Xu, Yi Liu

**Affiliations:** 1 Model Animal Research Center, Nanjing University, 12 Xuefu Road, Pukou District, Nanjing 210061 China; 2 Cambridge-Su Genomic Resource Center, Soochow University, Suzhou, Jiangsu 215123 China; 3 Department of Physiology, University of Texas Southwestern Medical Center, Dallas, TX 75390 USA

**Keywords:** Molecular biology, Genetics

Correction to: *Nature Communications* 10.1038/s41467-022-31715-4, published online 09 July 2022

The original version of this Article contained an error in Fig. 7b, in which the red bars should indicate the WT and the blue ones the mutant. This has been corrected in both the PDF and HTML versions of the Article.

