# Supplementary material for: Decoupling PER phosphorylation, stability and rhythmic expression from circadian clock function by abolishing PER-CK1 interaction
Source: Nat Commun. 2022 Jul 9;13:3991. doi: 10.1038/s41467-022-31715-4 (PMC9271041; doi:10.1038/s41467-022-31715-4)
Supplement: Supplementary file 1 — Supplementary Information [file 41467_2022_31715_MOESM1_ESM.pdf]

Supplementary Information

Title: Decoupling PER phosphorylation, stability and rhythmic expression from circadian clock function by abolishing PER-CK1 interaction

An et al.

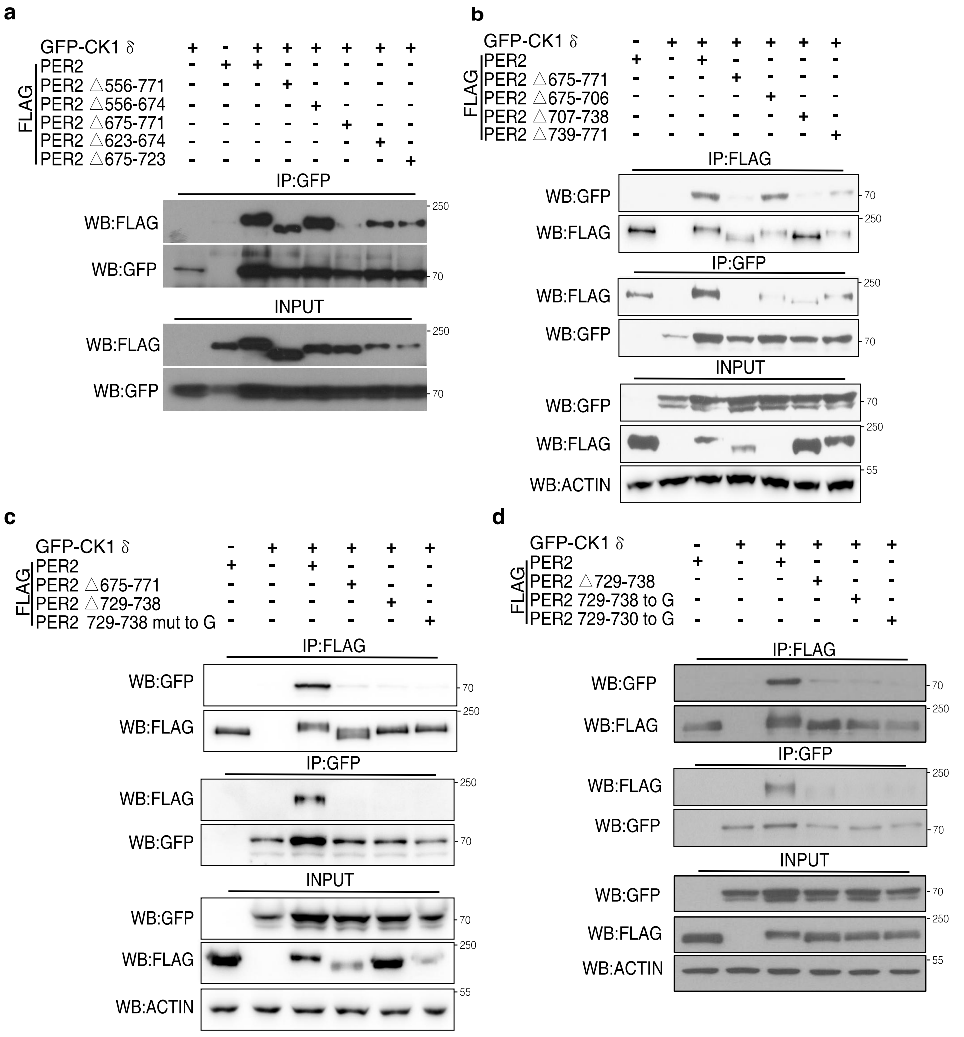

Supplementary Figure 1. PER2 729 and 730 amino acids play the critical role in PER2-CK1δ interaction.

Western blot analyses and immunoprecipitation assays show the difference of interaction strength between CK1 δ and hPER2 with indicated deletions or mutations.

(a) Analyses of hPER2 deletion constructs between 556 and 771 amino acids sites show that the CKIδ binding site is within 675 to 771 amino acid regions.

(b) Analyses of hPER2 deletion constructs between 675 and 771 amino acids show that the CKIδ binding site is within 707 to 738 amino acid regions.

(c) Analyses of hPER2 deletion constructs between 675-738 amino acids sites and 729-738 mutant constructs show that the CKIδ binding site is within 729 to 738 amino acid regions.

(d) Analyses of hPER2 mutation constructs between 729 and 738 amino acids sites show that the key CKIδ binding sites are 729 and 730 amino acids.

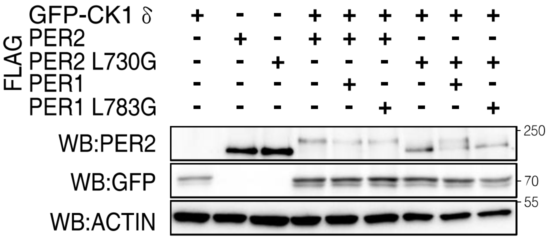

Supplementary Figure 2. PER1 L783G mutation abolishes the PER1's ability to promote CK1 phosphorylation of PER2 (L730G).

FLAG tagged PER1 with or without mutant, FLAG tagged PER2 with or without mutant and GFP tagged CK1δ were co-expressed in HEK293 cells (every 60-mm dish of cells was transfected with 2 μg of PER1 plasmid, 2 μg of PER2 plasmid and 1 μg of CK1δ plasmid). The levels of PER proteins and CK1δ were determined by western blot using anti-FLAG (sigma, 1:1000) and GFP (sigma, 1:1000) antibody, respectively.

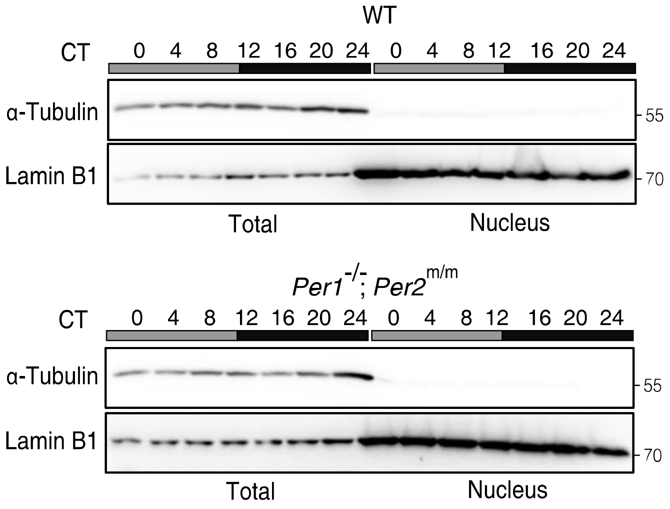

Supplementary Figure 3. Western blot identified WT and *Per1*$^{-/-}$; *Per2*$^{m/m}$ mice liver extract total Protein (α-Tubulin), nuclear protein (Lamin B1).

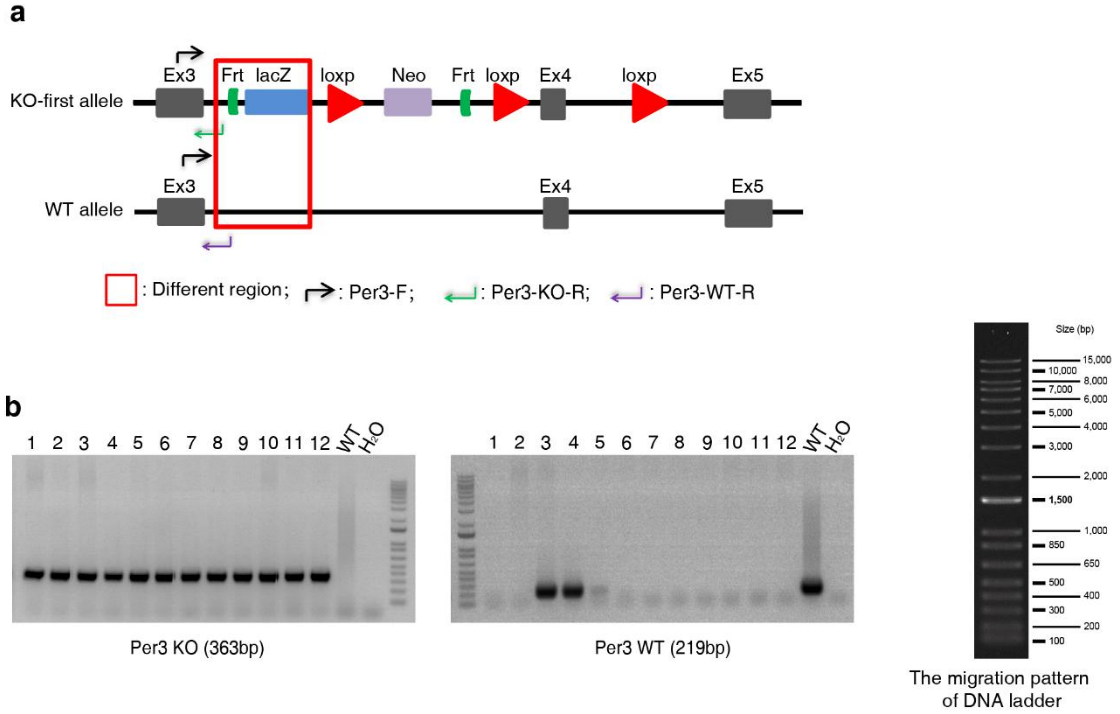

Supplementary Figure 4. Targeting strategy and Genotyping.

(**a**) Targeting strategies for *Per3* using EPD0602_1_G05 ES clone. The LacZ element contains a stop codon that also interrupts the transcription of *Per3*.

(**b**) PCR were used to confirm *Per3* knockout mice.
