# Supplementary material for: Decoupling PER phosphorylation, stability and rhythmic expression from circadian clock function by abolishing PER-CK1 interaction
Source: Nat Commun. 2022 Jul 9;13:3991. doi: 10.1038/s41467-022-31715-4 (PMC9271041; doi:10.1038/s41467-022-31715-4)
Supplement: Supplementary file 3 — Reporting Summary [file 41467_2022_31715_MOESM3_ESM.pdf]

nature portfolio

Corresponding author(s): Ying Xu,Yi Liu

Last updated by author(s): Jun 18, 2022

Reporting Summary

Nature Portfolio wishes to improve the reproducibility of the work that we publish. This form provides structure for consistency and transparency in reporting. For further information on Nature Portfolio policies, see our Editorial Policies and the Editorial Policy Checklist.

Statistics

For all statistical analyses, confirm that the following items are present in the figure legend, table legend, main text, or Methods section.

n/a	Confirmed	
☐	☒	The exact sample size (*n*) for each experimental group/condition, given as a discrete number and unit of measurement
☐	☒	A statement on whether measurements were taken from distinct samples or whether the same sample was measured repeatedly
☐	☒	The statistical test(s) used AND whether they are one- or two-sided<br>*Only common tests should be described solely by name; describe more complex techniques in the Methods section.*
☒	☐	A description of all covariates tested
☒	☐	A description of any assumptions or corrections, such as tests of normality and adjustment for multiple comparisons
☐	☒	A full description of the statistical parameters including central tendency (e.g. means) or other basic estimates (e.g. regression coefficient) AND variation (e.g. standard deviation) or associated estimates of uncertainty (e.g. confidence intervals)
☐	☒	For null hypothesis testing, the test statistic (e.g. *F*, *t*, *r*) with confidence intervals, effect sizes, degrees of freedom and *P* value noted<br>*Give P values as exact values whenever suitable.*
☒	☐	For Bayesian analysis, information on the choice of priors and Markov chain Monte Carlo settings
☒	☐	For hierarchical and complex designs, identification of the appropriate level for tests and full reporting of outcomes
☒	☐	Estimates of effect sizes (e.g. Cohen's *d*, Pearson's *r*), indicating how they were calculated

Our web collection on statistics for biologists contains articles on many of the points above.

Software and code

Policy information about availability of computer code

Data collection	DNBSEQ-T7 platform at BGI Genome Center, Shenzhen, China
Data analysis	RNA-seq<br>RNA gene expression profiles were analyzed using RSEM(version 1.2) with NCBI-annotation(version GRCm39).The genes expression levels were normalized with Z-scores, and visualized in a heatmap generated by using the R package pheatmap(version 4.1.0). DEGs were calculated using R package DEseq2(version 1.34.0). Functional annotation of DEGs was performed using The Database for Annotation, Visualization and Integrated Discovery (DAVID) (https://david.ncifcrf.gov/).<br><br>ChIP-seq<br>Chip seq profiles were analyzed using Bowtie2(version 2.3.4) for alignment and MACS(version 1.4.2)for peakcallig. Difference peaks were compared using R package DiffBind and annotated by ChIPseeker(version 1.30.0).<br><br>JTK_CYCLE(version 3.1) was used to decide rhythmic expressed transcripts. Statistical analyses were conducted using GraphPad Prism 8 software(GraphPad Software, Inc., CA, USA).

For manuscripts utilizing custom algorithms or software that are central to the research but not yet described in published literature, software must be made available to editors and reviewers. We strongly encourage code deposition in a community repository (e.g. GitHub). See the Nature Portfolio guidelines for submitting code & software for further information.

Data

Policy information about availability of data

All manuscripts must include a data availability statement. This statement should provide the following information, where applicable:

- Accession codes, unique identifiers, or web links for publicly available datasets
- A description of any restrictions on data availability
- For clinical datasets or third party data, please ensure that the statement adheres to our policy

All RNA-seq and ChIP-seq datasets is deposited in GEO repositoriest with the accession number PRJNA766147 (https://www.ncbi.nlm.nih.gov/sra/?term=PRJNA766147) and PRJNA766292(https://www.ncbi.nlm.nih.gov/sra/?term=PRJNA766292). Additional data supporting the findings of this work is available as supplemental files and source data are provided with this paper .

Field-specific reporting

Please select the one below that is the best fit for your research. If you are not sure, read the appropriate sections before making your selection.

☒ Life sciences ☐ Behavioural & social sciences ☐ Ecological, evolutionary & environmental sciences

For a reference copy of the document with all sections, see nature.com/documents/nr-reporting-summary-flat.pdf

Life sciences study design

All studies must disclose on these points even when the disclosure is negative.

Sample size	By convention and according to the standards in the circadian clock field (see e.g., Koike 2012, Science 338. DOI:10.1126/science.1226339).RNA-seq samples from WT and Per1-/-; Per2m/m were collected every 4 h over a 24 period with three replicates. Bmal1 ChIP-seq samples were collected every 4 h over a 24-h period for the WT and Per1-/-; Per2m/m mice without repeats. All other samples including animal numbers and WB independent repeats were indicated in the figures and figure legends.
Data exclusions	No data was excluded from the study.
Replication	All experiments used replicates which substantially agreed with each other.
Randomization	Randomization was not a component of our experimental design. To ensure comparable results within experiments RNA-seq, WB, and QPCR were performed simultaneously for different genotype mice with similar age under the SPF animal facility.
Blinding	Blinding was not performed as the experiments required knowledge of which condition and which genotype each sample belongs. Data collection and sample collection were not blinded as treatments were not a component of the study, rather was difference from genotypes.

Reporting for specific materials, systems and methods

We require information from authors about some types of materials, experimental systems and methods used in many studies. Here, indicate whether each material, system or method listed is relevant to your study. If you are not sure if a list item applies to your research, read the appropriate section before selecting a response.

Materials & experimental systems

n/a	Involved in the study	
☐	☒	Antibodies
☐	☒	Eukaryotic cell lines
☒	☐	Palaeontology and archaeology
☐	☒	Animals and other organisms
☒	☐	Human research participants
☒	☐	Clinical data
☒	☐	Dual use research of concern

Methods

n/a	Involved in the study	
☐	☒	ChIP-seq
☒	☐	Flow cytometry
☒	☐	MRI-based neuroimaging

Antibodies

Antibodies used	Immunoblot analysis was conducted following standard protocol with following antibodies: CK1δ(Abcam, ab85320?1:1000), PER1 (MBL, PM091,1:1000), PER2 (MBL, PM083,1:1000), PER2 S662(ABCAM,ab206377,1:1000),CLOCK (CST, 5157S,1:500), GFP (Sigma-Aldrich, G1544,1:1000), Flag (Sigma-Aldrich, F1804,1:1000), HA (Sigma-Aldrich, A2095,1:1000), Actin (Sigma-Aldrich, A5441,1:2000) and Tubulin (CST 5346s,1:3000). BMAl1 and CRY1 used in WB, CoIP and ChIP were previously described by Shi, G. et al. Dual roles of FBXL3 in the mammalian circadian feedback loops are important for period determination and robustness of the clock. Proc Natl Acad Sci U S A 110, 4750-5 (2013). Rabbit polyclonal BMAL1 and CRY1 antibodies were generated by Signalway Antibody using

synthetic peptides as antigen BMAL1: CSSSILGENPHIGIDMIDNDQGSSSPSNDEA; CRY1: CSQGSGILHYAHGDSQQ THSLKQGRSSAGTG and validated with mouse knockout samples.

Validation	All antibodies used in the western blots have been validated by the manufacturers, stating that the antibodies recognize endogenous levels of the target proteins in mouse tissues.<br>For all antibodies used in the ChIP-seq experiments, the manufacturers state that they recognize endogenous target proteins in both mouse tissues and human cell cultures and are suitable for ChIP-seq assays. Besides, our results demonstrated their strong activities.

Eukaryotic cell lines

Policy information about cell lines

Cell line source(s)	HEK293T cells were purchased from BLUEFBIO, Shanghai, China.
Authentication	All experiments in this study used low-passage cell cultures. The morphology of each cell line was consistent with images and descriptions on the ATCC website.
Mycoplasma contamination	All cell lines were periodically confirmed to be mycoplasma-free by using the PlasmoTest mycoplasma detection kit
Commonly misidentified lines (See ICLAC register)	No commonly misidentified line was used in this study.

Animals and other organisms

Policy information about studies involving animals; ARRIVE guidelines recommended for reporting animal research

Laboratory animals	Animal studies were conducted in the accredited SPF animal facility in the CAM-SU Genomic Resource Center and approved by the Animal Care and Use Committee of Soochow University, Suzhou, China (APYX2018-2, APYX2018-6). PER2V721G/L722G mice were generated by CRISPR/Cas9 technology on C57BL/6J background through the import of a mutant homologous repair template in the region corresponding to amino acids 721-722 site. The targeted guide RNA, Cas9 mRNA, and repair template were mixed and injected into two-cell stage embryos of C57BL/6J mice, and the embryos were then transplanted into a surrogate mouse uterus. The founders were characterized by genotyping and then crossed to C57BL/6J mice. Per3 knockout was made by the CAM-SU Genomic Resource Center. Per1 knockout mice was obtained from Jackson laboratory (Zheng et al., 2001). The F1 offspring were analyzed by DNA sequencing to confirm the germline transmission of intended mutations and then crossed to C57BL/6J, Per1-/-; or Per3-/- mice. In all experiments,male mice between 8 and 12 wk of age were used. In all experiments except the Free-running treatment, mice were housed in a room with controlled temperature of 21–23°C and humidity of 35–40% under a 12-h light/12-h dark (LD) cycle with free access to food and water.
Wild animals	This study did not use wild animals.
Field-collected samples	This study does not involved field-collected samples.
Ethics oversight	Animal studies were conducted in the accredited SPF animal facility in the CAM-SU Genomic Resource Center and approved by the Animal Care and Use Committee of Soochow University, Suzhou, China (APYX2018-2, APYX2018-6).

Note that full information on the approval of the study protocol must also be provided in the manuscript.

ChIP-seq

Data deposition

☒ Confirm that both raw and final processed data have been deposited in a public database such as GEO.

☐ Confirm that you have deposited or provided access to graph files (e.g. BED files) for the called peaks.

Data access links<br>*May remain private before publication.*	https://www.ncbi.nlm.nih.gov/bioproject/?term=prjna766292<br>All data was confirmed to access.
Files in database submission	BioSamples:SAMN21841486,SAMN21841487,SAMN21841488.<br>SRA:SRR16087528,SRR16087527,SRR16087526,SRR16087525,SRR16087524,SRR16087523,SRR16087522,SRR16087521,SRR16087520,SRR16087519,SRR16087518,SRR16087517,SRR16087531,SRR16087530,SRR16087529
Genome browser session (e.g. UCSC)	No longer applicable

Methodology

Replicates	Bmal1 ChIP-seq samples were collected every 4 h over a 24-h period for the WT and Per1-/-; Per2m/m mice.
Sequencing depth	ChIP-seq was performed on an Illumina MiSeq platform with PE 150-bp reads at the Novogene, Nanjing Sequencing Center, China.<br>Each sample is sequenced with no less than 6G of raw data.<br>Number of reads obtained from the final sequencing of the sample<br>WT-CT0 has total reads 19669493 (uniquely mapped reads 17452741);

WT-CT4 has total reads 16905337 (uniquely mapped reads 15245233);
WT-CT8 has total reads 26644208 (uniquely mapped reads 24398101);
WT-CT12 has total reads 19019586 (uniquely mapped reads 17359176);
WT-CT16 has total reads 22332762 (uniquely mapped reads 20706937);
WT-CT20 has total reads 17021447 (uniquely mapped reads 15898032);
WT-CT24 has total reads 16076585 (uniquely mapped reads 14674707);
Mutation-CT0 has total reads 17092160 (uniquely mapped reads 14928293);
Mutation-CT4 has total reads 13223081 (uniquely mapped reads 12319945);
Mutation-CT8 has total reads 20830666 (uniquely mapped reads 19730807);
Mutation-CT12 has total reads 15138774 (uniquely mapped reads 14106310);
Mutation-CT16 has total reads 18791121 (uniquely mapped reads 11150651);
Mutation-CT20 has total reads 20407375 (uniquely mapped reads 18805396);
Mutation-CT24 has total reads 19952255 (uniquely mapped reads 18330137);
Input has total reads 25084770 (uniquely mapped reads 24304634);

Antibodies

BMAL1 antibody

Peak calling parameters

-f SAM -p 1e-5 -w -S -g mm

Data quality

The quality of the total DNA was determined using an xxxx and reads were quality checked with FastQC v0.11.8. Number of peaks at FDR 5% and above 5-fold enrichment:
WT-CT0 has 0; WT-CT4 has 211; WT-CT8 has 405; WT-CT12 has 202; WT-CT16 has 0; WT-CT20 has 0; WT-CT24 has 0;
Mutation-CT0 has 70; Mutation-CT4 has 459; Mutation-CT8 has 34; Mutation-CT12 has 0; Mutation-CT16 has 0; Mutation-CT20 has 0; Mutation-CT24 has 0.

Software

Chip seq profiles were analyzed using Bowtie2(version 2.3.4) for alignment and MACS(version 1.4.2)for peakcallig. Difference peaks were compared using R package DiffBind and annotated by ChIPseeker. BMAL1 Chip-seq data described in this study are deposited in the BioProject with the accession number PRJNA766292
